# Reevaluating the Potential of a Vanilla Transformer Encoder for Unsupervised Time Series Anomaly Detection in Sensor Applications

**DOI:** 10.3390/s25082510

**Published:** 2025-04-16

**Authors:** Chan Sik Han, HyungWon Kim, Keon Myung Lee

**Affiliations:** 1 Department of Computer Science, Chungbuk National University, Cheongju 28644, Republic of Korea; chatterboy@chungbuk.ac.kr; 2 Department of Electronics Engineering, Chungbuk National University, Cheongju 28644, Republic of Korea; hwkim@chungbuk.ac.kr

**Keywords:** time series anomaly detection, unsupervised learning, vanilla transformer encoders, careful design choices

## Abstract

Sensors generate extensive time series data across various domains, and effective methods for detecting anomalies in such data are still in high demand. Unsupervised time series anomaly detection provides practical approaches to addressing the challenges of collecting anomalous data. For effective anomaly detection, a range of deep-learning-based models have been explored to handle temporal patterns inherent in time series data. In particular, Transformer encoders have gained significant attention due to their ability to efficiently capture temporal dependencies. Various studies have attempted the architectural improvements of Transformer encoders to address the inherent complexity of time series data analysis. Unlike the previous studies, this work demonstrates that a vanilla Transformer encoder-based framework remains yet a competitive model for time series anomaly detection. Instead of architectural modification of the Transformer encoder, we identify key design choices and propose an asymmetric autoencoder-based framework incorporating those design choices with a vanilla Transformer encoder and a linear layer decoder. The proposed framework has been evaluated on a range of unsupervised time series anomaly detection benchmarks, and the experimental results show that it achieves performance that is either superior or competitive compared to state-of-the-art models.

## 1. Introduction

Time series anomaly detection is used to identify unusual patterns or events in time series data [1]. This is an important problem across many domains like finance, manufacturing, and healthcare [2]. Due to its importance, this problem has been a focus of research for several decades. Initially, statistical methods [3,4,5,6] and machine-learning methods [7,8,9,10] were studied. Recently, with advancements in deep learning, there has been increasing interest in applying deep-learning models to time series anomaly detection [11]. In particular, unsupervised time series anomaly detection has attracted significant attention due to the challenges of collecting abnormal data in real-world scenarios, such as the rarity of anomalies and the diversity of anomaly patterns [12,13,14,15].

In unsupervised time series anomaly detection, numerous models have been proposed. Some studies have focused on capturing temporal dependencies using Recurrent Neural Networks (RNNs) and Long Short-Term Memory networks (LSTMs) [16,17,18], while others have used Convolution Neural Networks (CNNs) and Temporal Convolution Networks (TCNs) to extract temporal features locally and hierarchically [19,20,21]. Additionally, Graph Neural Networks have been employed to model complex relationships in multivariate time series data [22,23,24]. Generative models, such as Variational Autoencoders, Generative Adversarial Networks, Normalizing Flows, and Diffusion models, have also been explored. These models detect anomalies by analyzing reconstruction errors or deviations from learned data distributions [25,26]. Autoencoders, in particular, are widely adopted due to their simplicity and effectiveness in capturing temporal features [27].

Transformers outperformed previous models in natural language processing [28]. Since then, they have been successfully applied across diverse domains, proving their effectiveness. The success of Transformers has driven extensive research into their application to unsupervised time series anomaly detection [29,30,31,32]. In particular, Transformer encoders have gained attention for their ability to efficiently capture temporal dependencies. However, due to the inherent complexity of time series data, various studies have attempted to improve Transformer encoders by architectural modifications such as a prior-association branch [33] and a gated memory module [34]. These architectural improvements have led to notable performance gains on various benchmarks [29,33,34,35].

This study demonstrates that effective anomaly detection could be achieved without such structural modifications solely through careful design choices within a vanilla Transformer encoder-based asymmetric autoencoder framework. To substantiate this, we identify key design choices, which include dataset-level and sub-dataset-level normalizations (for preprocessing raw time series data), time series segment generation, segment-level normalization and denormalization, time series segment embedding, and a simple decoder for asymmetric autoencoder realization. Additionally, we propose an autoencoder-based anomaly detection framework that consists of a vanilla Transformer encoder and a linear layer decoder, incorporating key design choices. The proposed framework has been assessed on several widely used benchmarks for unsupervised time series anomaly detection. The experimental results show that the proposed framework achieves a performance superior to or comparable to recent Transformer encoder-based models with architectural modifications. These findings reveal the often-overlooked potential of vanilla Transformer encoders for unsupervised time series anomaly detection. The code for the proposed framework, along with its pretrained weights for the benchmark datasets, is publicly available at https://github.com/chatterboy/revisitVanillaTransEncUnsupTSAD (accessed on 13 April 2025).

## 2. Related Work

In unsupervised time series anomaly detection, deep-learning-based anomaly detection models have been actively studied [36]. In particular, Transformer encoder-based anomaly detection models have gained significant attention due to their ability to efficiently capture the temporal patterns inherent in time series data [37]. While Transformer encoder-based models have proven effective, they still encounter challenges stemming from the complex nature of time series data. These challenges include inter-variable dependencies, distribution shifts due to non-stationarity, sparsely occurring anomalies, and diverse anomaly patterns [38].

To address these challenges, previous studies have mainly attempted to modify the architectural features of transformer encoders. GTA [24] incorporated a graph neural network to effectively capture inter-variable dependencies in multivariate time series data. Anomaly Transformer [33] introduced an additional branch to capture associations in normal data while considering the inductive bias of anomalous data. DCdetector [39] utilized a two-tower architecture that simultaneously learns patch-wise and in-patch association representations to enhance the representation capability of normal data. MEMTO [34] leveraged a gated memory module to distinguish normal data and abnormal data based on the prototypical features of normal data. AnomalyLLM [35] employed a teacher-student framework in which the student network is trained to replicate the features of a teacher network, adapted from a pretrained large language model.

While previous studies have mainly concentrated on enhancing Transformer encoder architectures for effective anomaly detection, this study takes a different approach. Instead of modifying the architecture, we explore how effective unsupervised time series anomaly detection can be achieved purely through strategic design choices applied to a vanilla Transformer encoder-based asymmetric autoencoder framework.

## 3. Materials and Methods

**Figure 1:**
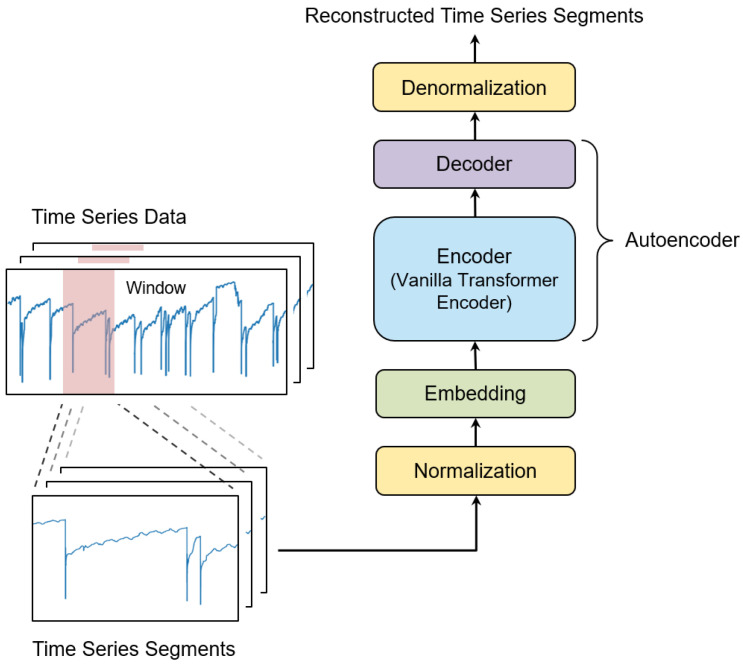
Overview of the proposed framework for unsupervised time series anomaly detection. This framework is designed to reconstruct input time series segments and consists of several key modules: preprocessing time series data, generating time series segments, segment-level normalization and denormalization, time series segment embedding, and an asymmetric encoder-decoder.

This work aims to revisit the potential of a vanilla Transformer encoder in unsupervised time series anomaly detection. To evaluate the capabilities of a vanilla Transformer encoder, we propose an autoencoder-based framework equipped with a vanilla Transformer encoder and a linear layer decoder (see Figure 1). This framework makes use of a simple architecture while enabling a vanilla Transformer encoder to effectively capture temporal dependencies. This framework takes time series segments generated from (preprocessed) time series data as input and reconstructs the taken time series segments. The proposed framework consists of the following modules: preprocessing time series, generating time series segments, segment-level normalization-denormalization, time series segment embedding, and an asymmetric encoder-decoder.

### 3.1. Preprocessing Time Series Data

Preprocessing plays a vital role in the development of a deep-learning framework. We are concerned with normalization techniques for preprocessing in the context of unsupervised learning-based time series anomaly detection. They are used to convert the scale of time series data into a specified interval, which usually significantly impacts the performance of the anomaly detection process.

Representative normalization techniques include min-max normalization and z-score normalization. Let $X\in R^{T\times V}$ represent time series data, where $x_{ij}$ denotes the value at the *i*-th time step of the *j*-th variable. Its normalized time series data $\tilde{X}\in R^{T\times V}$ can be expressed in $\tilde{X}=\phi \left(X\right)$, where ϕ(·) is a normalization method which is defined as follows:

(1) $$\phi^{\min-\max}\left(x_{i,j}\right)=\frac{x_{i,j}-\min_{k}\left\{x_{i,k}\right\}}{\max_{k}\left\{x_{i,k}\right\}-\min_{k}\left\{x_{i,k}\right\}}\;\min-\max\;\mathrm{normalization}$$

(2) $$\phi^{z-\mathrm{score}}\left(x_{i,j}\right)=\frac{x_{i,j}-\mu_{\cdot ,j}}{\sigma_{\cdot ,j}}\;z-\mathrm{score}\;\mathrm{normalization}$$

where $\mu_{\cdot ,j}$ and $\sigma_{\cdot ,j}$ represent the mean and standard deviation of the *j*-th variable, respectively. As the decision choices for the normalization strategy, the framework is designed to examine the impact of three normalization strategies: no normalization, min-max normalization, and z-score normalization. The best choice is determined based on performance evaluations for each dataset.

Some datasets are composed of multiple sub-datasets (see Table 1 and Table 2). For instance, the multivariate dataset MSL consists of 27 sub-datasets, and the univariate dataset ABP consists of 42 sub-datasets. For datasets consisting of multiple sub-datasets, we apply two levels of normalization sequentially: first at the sub-dataset level, followed by the dataset level, with three normalization options available at each level. Sub-dataset-level normalization is performed on each sub-dataset individually, while dataset-level normalization is applied across all sub-datasets as a whole. The total nine combinations of normalization methods for the sub-dataset level and the dataset level are examined to select the best one. At inference time, parameters such as min, max, mean, and standard deviation computed during training are utilized.

### 3.2. Generating Time Series Segments

Time series datasets often consist of long sequences dynamically collected in real-world environments. These characteristics can make it difficult to directly feed raw time series data into an outlier detection model. To address this, techniques for generating fixed-length segments from the original data are employed, facilitating easier analysis and processing by the model.

The windowing technique is a commonly used method for generating time series segments in unsupervised anomaly detection for time series data. It involves copying fixed-length segments from an original time series data at regular intervals using a sliding window. Varying the window length and stride enables the generation of different sets of time series segments from the data. The process of generating a full set of time series segments from preprocessed data using the windowing technique can be outlined as follows:

(3) $$x=W(\tilde{X})$$

where W(·) is a window function, $W:R^{T\times V}\to R^{N\times L\times V}$, x and $\tilde{X}$ denote the time series segment and its preprocessed dataset, respectively.

The optimal window length and stride size for capturing temporal dependencies effectively can vary across datasets. To address this, we adopt window lengths and stride sizes tailored to each dataset. Furthermore, stride size can be applied differently during training and evaluation. During evaluation, the stride size matches the window length, following the non-overlapping approach used in prior studies such as [17,33,34,39]. In contrast, during training, the stride size is selected based on whether an overlapping or non-overlapping approach is more effective for capturing temporal dependencies. This approach ensures efficient temporal pattern extraction during training.

### 3.3. Segment-Level Normalization and Denormalization

Real-world time series data are often non-stationary, with statistical properties that change over time. This non-stationarity poses significant challenges in extracting meaningful temporal features. Since time series segments are derived from non-stationary data, they inherently retain this non-stationarity. To address these challenges, we use a segment-level normalization and denormalization technique designed to effectively capture temporal dependencies in non-stationary time series segments. As shown in Figure 1, the normalization is applied to time series segments prior to their embedding. The embedded time series segments pass through the encoder and the corresponding time series segments are reconstructed by the decoder. The reconstructed one is denormalized into the scale of original time series segments.

We employ a reversible instance normalization method [40], originally proposed for time series forecasting to address distribution shifts between training and testing datasets, for normalizing and denormalizing time series segments. Given batched time series segments $x^{\prime }\in R^{B\times L\times V}$ with batch size *B*, the sequence length *L* and the number of variables *V*, the normalization method is applied to multivariate time series segments as follows:

(4) $${\tilde{x}}_{i,:,j}=\alpha_{j}\left(\frac{x_{i,:,j}^{\prime }-\mu_{i,j}}{\sigma_{i,j}}\right)+\beta_{j}$$

where $\tilde{x}\in R^{B\times L\times V}$ represents the normalized time series segments, $\mu_{i,j}$ and $\sigma_{i,j}$ denote the mean and standard deviation of the data points of the *j*-th variable within a time series segment *i*, and $\alpha_{j}$ and $\beta_{j}$ are learnable parameters used for scaling and shifting of the *j*-th variable. The normalization has the effect of alleviating distributional shifts and scale variations caused by non-stationarity [40]. It is used with the expectation of making the time series stationary. The normalization method is applied in a segment-wise manner to the time series, where the mean and variance are computed and used for each segment.

The encoder and decoder are trained to reconstruct the normalized time series segments at the output of the decoder. For normalized time series segments reconstructed by the decoder, denormalization is carried out as follows:

(5) $${\hat{x}}_{i,:,j}=\sigma_{i,j}\left(\frac{{\bar{x}}_{i,:,j}-\beta_{j}}{\alpha_{j}}\right)+\mu_{i,j}$$

where $\hat{x}\in R^{B\times L\times V}$ represents the final denormalized reconstructed time series segments produced by the model, while $\bar{x}$ represents the normalized reconstructed time series segments generated by the decoder.

### 3.4. Time Series Segment Embedding

Time series segment embedding is needed to transform time series segments into a format suitable for the proposed autoencoder-based architecture, which consists of the encoder and decoder shown in Figure 1. The embedding module converts the data points of dimension *V* within time series segments into real-valued vectors of dimension *F*. To capture both individual data-point information and short-term temporal dependencies within time series segments, we use a 1D convolutional layer for embedding, i.e., the embedding method generates an embedding vector **e** by considering individual data points along with their neighboring data points for a time series segment $\bar{x}$.

(6) $$e=\mathrm{Conv}1d(\tilde{x})$$

where $e\in R^{B\times L\times F}$ is a batch of *B* sequences, where each sequence consists of *L* embedding vectors with a feature dimension of *F*. The 1D convolutional layer consists of *F* kernels, each with a length of 3, a stride of 1, and no bias term, to generate an embedding vector of dimension *F*. Zero padding is applied to ensure that the sequence length of the embedded time series segment matches that of the original time series segment.

Additionally, we choose not to use positional encoding (PE), which is commonly employed in Transformers to incorporate positional information for extracting position-based features. This decision is based on two key considerations. First, positional encoding works by injecting additional positional information into the data. However, this process may lead the model to interpret the sequential flow of the data based on the positional encoding rather than its original order. As a result, the model may fail to properly learn the natural sequential relationships in the data. In reconstruction-based methods, preserving the original sequence is crucial, and such distortions can negatively impact reconstruction performance. Second, a reconstruction model, such as an autoencoder, aims to reproduce the input data as accurately as possible. Adding positional encoding introduces new patterns to the data, which might cause the model to learn these patterns inappropriately or to overlook essential structural information from the original data by overemphasizing positional differences. This may impair the model’s reconstruction performance, resulting in degrading its anomaly detection capabilities. To assess the impact of positional encoding, we have conducted an ablation study as part of our analysis (see Section 3.4).

### 3.5. Asymmetric Autoencoder

Autoencoders are widely used in unsupervised time series anomaly detection because of their simplicity and capability to reconstruct input data. An autoencoder consists of two components: an encoder and a decoder. The encoder extracts features from the input data while the decoder reconstructs the input data from these extracted features. According to Wang et al. [41], an excessively powerful decoder can impede the encoder’s ability to effectively capture meaningful features. This happens because the powerful decoder can be trained to nearly perfectly reconstruct the input data without strongly relying on useful latent representations produced by the encoder. Rather than encouraging the encoder to learn meaningful representations, the decoder may instead memorize trivial patterns, resulting in poor generalization.

To mitigate this issue, we propose an asymmetric autoencoder design where the encoder is more complex and parameter-rich while the decoder is kept simpler with fewer parameters. This asymmetry ensures that the decoder focuses solely on reconstruction, allowing the encoder to effectively capture rich and informative temporal features from the time series segments. Specifically, we use a vanilla Transformer encoder for the encoder and a linear layer for the decoder.

Given a batch of embedding vector sequences $e\in R^{B\times L\times F}$, a vanilla Transformer encoder produces a batch of hidden vector sequences $h\in R^{B\times L\times F}$ as follows:

(7) h=VanillaTransformerEncoder(e)

where h is the output of the last encoder block in a vanilla Transformer encoder. Then, a decoder reconstructs normalized time series segments for given hidden vector sequences.

The decoder, implemented as a linear layer, transforms each hidden vector $h\in R^{F}$ from the encoder into a data-point $\bar{x}\in R^{V}$ within a normalized time series segment, as follows:

(8) $$\bar{x}=Wh$$

where $W\in R^{V\times F}$ is a weight matrix for the decoder. The decoder processes each hidden vector generated by the encoder to reconstruct the normalized time series segment corresponding to the input time series segment provided to the encoder.

### 3.6. Training

The proposed framework is an autoencoder-based model designed to reconstruct given time series segments. To achieve this, we employ a reconstruction-based training approach that minimizes the discrepancy between the input and the reconstructed output. Specifically, the model is trained to reduce the difference between the input time series segments and the corresponding reconstructed segments generated by the framework. Let $x^{\prime }\in R^{B\times L\times V}$ represent the input time series segments, and let $\hat{x}\in R^{B\times L\times V}$ denote the reconstructed segments. The model is optimized using the following loss function:

(9) $$\ell \left(x^{\prime },\hat{x}\right)=\frac{1}{B}\sum_{b=1}^{B}{∥x_{b}^{\prime }-{\hat{x}}_{b}∥}_{2}^{2}$$

where *B* is batch size, and $x_{b}^{\prime }$ and ${\hat{x}}_{b}$ denote the *b*-th segments of $x^{\prime }$ and $\hat{x}$, respectively.

### 3.7. Evaluation

We use an evaluation protocol commonly used in reconstruction-based methods. The protocol consists of three steps: first, computing anomaly scores; second, predicting whether each timestamp is normal or abnormal; and third, evaluating the predictions using standard evaluation metrics.

Various methods exist for computing anomaly scores in reconstruction-based approaches. We utilize the anomaly scoring method proposed in the literature [33,34,35,39]. For a given data-point $x_{t}^{\prime }\in R^{V}$ from the batched time series segments $x^{\prime }$ and its reconstructed counterpart ${\hat{x}}_{t}\in R^{V}$ produced by the trained model, the anomaly score $s_{t}$ at timestamp *t* is computed as follows:

(10) $$s_{t}={∥x_{t}^{\prime }-{\hat{x}}_{t}∥}_{2}$$

To predict the anomaly status ${\hat{y}}_{t}$ at each timestamp *t* within the time series data $\tilde{X}$, we compute it as follows:

(11) $${\hat{y}}_{t}=\left\{\begin{array}{c} 1,\;s_{t}\geq \theta \\ 0,\;\mathrm{otherwise} \end{array}\right.$$

Here, ${\hat{y}}_{t}$ represents the anomaly status prediction, where ${\hat{y}}_{t}\in 0,1$, and θ is the threshold used to determine whether a data-point is anomalous or normal. The threshold is a critical parameter for accurate predictions. Among the various methods available for threshold selection, we adopt the approach used in the previous comparative studies [33,34,39].

To assess model performance, we employ several metrics. Dor experiments on multivariate benchmarks, we use Precision, Recall, and F1 scores. Given the ground truth anomaly labels $y\in {\left\{0,1\right\}}^{T}$ and the predicted anomaly labels $\hat{y}\in {\left\{0,1\right\}}^{T}$, these metrics are defined as follows:

(12) $$\mathrm{Precision}=\frac{\mathrm{TP}}{\mathrm{TP}+\mathrm{FP}}$$

(13) $$\mathrm{Recall}=\frac{\mathrm{TP}}{\mathrm{TP}+\mathrm{FN}}$$

(14) $$F1\;\mathrm{score}=2\times \frac{\mathrm{Preicison}\times \mathrm{Recall}}{\mathrm{Precision}+\mathrm{Recall}}$$

Here, TP (True Positive) denotes data points correctly identified as anomalies, while TN (True Negative) refers to data points accurately classified as normal. FP (False Positive) represents instances mistakenly detected as anomalies despite being normal, whereas FN (False Negative) refers to data points incorrectly classified as normal when they are actually anomalies. Additionally, we apply Point Adjustment (PA) [42], a post-processing technique that considers an entire anomalous segment correctly identified if at least one data-point within the segment is detected as anomalous. The PA-based metrics are employed to ensure a fair comparison between the proposed model and recent models on multivariate benchmarks [33,34,35,39].

While the PA-based metrics capture real-world anomaly detection scenarios [42], they have recognized limitations in evaluating model performance [43,44]. To overcome these shortcomings, we also incorporate affiliation metrics [45]. Unlike Precision, Recall, and the F1 score, which assess performance at the data-point level, affiliation metrics evaluate performance at the segment (event) level. Given the ground truth anomaly segments $e=\{e_{1},e_{2},...,e_{n}\}$ and the predicted anomaly segments $\hat{e}=\left\{{\hat{e}}_{1},{\hat{e}}_{2}...,{\hat{e}}_{m}\right\}$, affiliation Precision, affiliation Recall, and affiliation F1 score are defined as follows:

(15) $$\mathrm{affiliation}\;\mathrm{Precision}=\frac{1}{\left|S\right|}\sum_{i\in S}P_{{\mathrm{precision}}_{i}}\left(e_{i}\right)$$

(16) $$\mathrm{affiliation}\;\mathrm{Recall}=\frac{1}{n}\sum_{i=1}^{n}P_{{\mathrm{recall}}_{i}}\left(\left\{\hat{e}\cap I_{i}\right\}\right)$$

(17) $$\mathrm{affiliation}\;F1\;\mathrm{score}=2\times \frac{\mathrm{affiliation}\;\mathrm{Precision}\times \mathrm{affiliation}\;\mathrm{Recall}}{\mathrm{affiliation}\;\mathrm{Precision}+\mathrm{affiliation}\;\mathrm{Recall}}$$

Here, *S* represents ground truth anomaly segments that have at least one corresponding predicted anomaly segment. $P_{{\mathrm{precision}}_{i}}$ indicates whether the predicted anomaly segments assigned to the *i*-th ground truth anomaly segment correctly predict it, while $\left\{\hat{e}\cap I_{i}\right\}$ denotes the predicted anomaly segments associated with the *i*-th ground truth anomaly segment. Additionally, $P_{{\mathrm{recall}}_{i}}$ represents whether the *i*-th ground truth anomaly segment correctly matches its corresponding predicted anomaly segments. The affiliation metrics are employed for comparison on univariate benchmarks.

## 4. Comparative Experiments and Results

### 4.1. Benchmark Datasets

The benchmark datasets utilized in this study comprise real-world time series data collected from various domains, including space environments, industrial systems, and healthcare. These datasets inherently contain anomalies, which are identified by domain experts rather than through predefined synthetic distortions. To better characterize the nature of anomalies within these datasets, we classify them into three types [36]:

- Point anomaly: Individual data points that significantly deviate from expected values.
- Contextual anomaly: Data points that appear normal in isolation but are deemed anomalous when viewed within a specific context.
- Collective anomaly: A group of data points that, when considered together, exhibit abnormal behavior, even if individual points appear normal.

Each benchmark comprises a combination of these anomaly types, depending on its inherent characteristics. These datasets are employed to assess the proposed model, demonstrating its effectiveness in detecting anomalies across diverse real-world scenarios.

#### 4.1.1. Multivariate Datasets

We use six multivariate time series anomaly detection benchmarks, as described in Table 1. The MSL [16] contains telemetry data with labeled anomalies from the Mars Science Laboratory rover. The SMAP [16] includes telemetry and anomaly labels from the Soil Moisture Active Passive satellite. The SMD [25] is a labeled multivariate time series dataset containing metrics like CPU and memory usage from 28 server machines over 5 weeks. The PSM [46] features anonymized server metrics from eBay spanning 13 weeks of training and 8 weeks of testing. The GECCO [38] focuses on drinking water quality monitoring, providing multivariate time series data with physical and chemical properties. The SWAN-SF [38] includes multivariate time series data on extreme space weather conditions for space weather analysis.

**Table 1:** Description of multivariate time series anomaly detection benchmark datasets. The symbol “#” denotes “number of”.

	# Sub-Datasets	# Dimensions	# Training Data Points	# Test Data Points	Anomaly Ratio (%) ^†^
MSL	27	55	58,317	73,729	10.53
SMAP	54	25	138,004	435,826	12.84
SMD	28	38	708,405	708,420	4.16
PSM	1	25	132,481	87,841	27.76
GECCO	1	9	69,260	69,261	1.05
SWAN-SF	1	38	60,000	60,000	32.60

^†^ The anomaly ratio in the test set.

#### 4.1.2. Univariate Datasets

We employ nine univariate time series anomaly detection benchmark datasets, described in Table 2. These datasets are derived from the UCR Anomaly Archive [47], following the categorization by Goswami et al. [48]. The UCR Anomaly Archive consists of 250 sub-datasets, each originating from a specific domain. Goswami et al. [48] grouped these sub-datasets into nine distinct domains: ABP, Acceleration, Air Temperature, ECG, EPG, Gait, NASA, Power Demand, and RESP. To construct the datasets, we concatenated all sub-datasets within each domain.

**Table 2:** Description of univariate time series anomaly detection benchmark datasets.

	# Sub-Datasets	# Training Data Points	# Test Data Points	Anomaly Ratio (%) ^†^
ABP	42	1,036,746	1,841,461	0.37
Acceleration	7	38,400	62,337	1.71
Air Temperature	13	52,000	54,392	0.82
ECG	91	1,795,083	6,047,314	0.38
EPG	25	119,000	410,415	0.45
Gait	33	1,157,571	2,784,520	0.38
NASA	11	38,500	86,296	0.86
Power Demand	11	197,149	311,629	0.61
RESP	17	868,000	2,452,953	0.12

^†^ The anomaly ratio in the test set.

### 4.2. Baselines

To assess the effectiveness of the proposed framework, we compare it with recent advanced deep-learning methods for unsupervised time series anomaly detection. For multivariate datasets, the baseline methods include Anomaly Transformer [33], DCdetector [39], MEMTO [34], and AnomalyLLM [35]. For univariate datasets, the comparisons feature TS-TCC [49], THOC [17], NCAD [50], and AnomalyLLM [35].

### 4.3. Implementation Details

The proposed framework was implemented using Python 3.8.19 and PyTorch 2.1.0. Training and evaluation were conducted on a single NVIDIA A100 GPU, utilizing the Adam optimizer [51]. The hyperparameter configurations for the multivariate and univariate datasets are detailed in Table A1 and Table A2, respectively. Key hyperparameters were determined through a grid search, while other parameters were set to commonly used default values.

### 4.4. Main Results

Table 3 presents the F1 scores comparing the proposed vanilla Transformer encoder-based model with state-of-the-art time series anomaly detection methods, including Anomaly Transformer [33], DCdetector [39], MEMTO [34], and AnomalyLLM [35], across the six multivariate time series datasets.

The results for the proposed model were obtained following the aforementioned experimental procedures, while the results for the other models were taken from their respective publications. The findings reveal that the proposed model achieved competitive performance across most datasets, with superior results on some. Specifically, it recorded the highest F1 scores of 0.975 and 0.920 on SMAP and GECCO, respectively. For SMD, PSM, and SWAN-SF, it achieved the second-highest performance, following AnomalyLLM [35]. In particular, on GECCO, the proposed model outperformed both DCdetector [39] and AnomalyLLM [35] by a substantial margin.

These results highlight that, despite its relatively simple architecture, the proposed model delivers performance comparable to or better than more advanced models. Through thoughtful design choices, the vanilla Transformer encoder-based model effectively captures the temporal dependencies inherent in time series data. Further details on the experimental results are provided in Table A3.

Table 4 presents the Affiliation F1 scores comparing the proposed vanilla Transformer encoder-based model with state-of-the-art time series anomaly detection methods, including TS-TCC [49], THOC [17], NCAD [50], and AnomalyLLM [35], across the nine univariate time series datasets. The results of the proposed model were obtained using the outlined experimental procedures, while those for the other models were sourced from the experiments in AnomalyLLM [35].

The findings show that, with few exceptions, the proposed model demonstrated competitive or superior performance in most datasets. Notably, it achieved the highest scores of 0.966, 0.802, and 0.763 on Acceleration, ECG, and RESP, respectively. For datasets such as ABP, Air Temperature, Gait, NASA, Power Demand, and Average, the model delivered the second-best performance. Although the proposed model showed a noticeable performance gap compared to AnomalyLLM [35] on ABP and EPG, it performed competitively or even better on the remaining datasets.

As with the multivariate dataset experiments, these results confirm that the proposed model, despite its relatively simple architecture, achieves performance comparable to or exceeding that of advanced methods. This shows that with thoughtful design choices, a vanilla Transformer encoder-based model can effectively capture temporal features. Additional details on the experimental results are provided in Table A4.

Additionally, we conducted further comparative experiments on the proposed model using various evaluation metrics on multivariate benchmarks. We utilized accuracy and PA-based F1-score, along with Affiliation Precision, Affiliation Recall, Range-AUC-ROC [52], Range-AUC-PR [52], VUS-ROC [52], and VUS-PR [52]. Unlike accuracy and F1 score, which measure performance at the data-point level, the other metrics (Affiliation Precision, Affiliation Recall, Range-AUC-ROC, Range-AUC-PR, VUS-ROC, and VUS-PR) assess performance at the event (anomalous segment) level. These event-level evaluation metrics are intended to offer a more comprehensive and robust assessment of model performance in time series anomaly detection.

Table 5 presents the evaluation results of the proposed model alongside compared models across the benchmarks. The results for the proposed model are obtained from our experiments, while those for the compared models are based on the reported performance of DCdetector [39]. Our findings indicate that the relative superiority of each model depends on the specific evaluation metric used.

First, we observed that a model that performs well on data-point-level metrics does not necessarily achieve the best results on event-level metrics. For example, in the MSL dataset, DCdetector [39] demonstrated the highest performance in data-point-level metrics but did not achieve the top scores in certain event-level metrics. Similarly, in the SMAP dataset, while the proposed model performed best overall in data-point-level metrics, it achieved the highest performance in only two out of six event-level metrics.

Second, in event-level evaluation, model rankings fluctuate considerably depending on the selected metric. For instance, in the MSL dataset, the leading model alternates between the proposed model and DCdetector [39], depending on the evaluation metric. Likewise, in the SMAP dataset, the ranking varies among the proposed model, DCdetector [39], and Anomaly Transformer [33]. These findings highlight the importance of using diverse evaluation metrics for a more comprehensive assessment of model performance.

Finally, in the PSM dataset, the proposed model consistently outperformed the compared models across both data-point-level and event-level metrics.

In conclusion, our experiments show that the proposed model delivers competitive performance compared to the latest models, Anomaly Transformer [33] and DCdetector [39]. This underscores that effective unsupervised anomaly detection can be accomplished solely through thoughtful design choices, without requiring architectural modifications to the Transformer encoder-based autoencoder model.

### 4.5. Ablation Study

We conducted an ablation study to evaluate the impact of the key design principles underlying the proposed framework. These principles include: (1) segment-level normalization and denormalization, (2) the use of positional encoding, and (3) preprocessing of time series data.

#### 4.5.1. Segment-Level Normalization and Denormalization

To evaluate the effectiveness of segment-level normalization and denormalization, we assessed the model’s performance with and without RevIN. For this analysis, three multivariate datasets (MSL, SMAP, and SMD) and three univariate datasets (ECG, Gait, and RESP) were selected. The F1 score was used as the evaluation metric for the multivariate datasets, while the affiliation F1 score was applied for the univariate datasets.

Table 6 shows the F1 scores for the multivariate datasets, and Table 7 presents the affiliation F1 scores for the univariate datasets. In the experiments with the multivariate datasets, incorporating RevIN consistently led to improved performance across all datasets. Similarly, in the univariate dataset experiments, RevIN generally achieved superior performance. These findings demonstrate that employing RevIN can enhance the performance of both multivariate and univariate anomaly detection tasks.

RevIN has been developed to address distribution shift issues that occur between training and testing datasets in time series forecasting. In this study, we used RevIN not only to mitigate distribution shifts between training and testing datasets but also to alleviate distribution shifts between time series segments in window-based anomaly detection. Such segment-level distribution shifts can prevent a model from effectively learning general temporal patterns during training. By addressing this issue, RevIN facilitates the efficient capture of general temporal patterns.

#### 4.5.2. Positional Encoding

The analysis revealed that incorporating positional encoding (PE) does not consistently improve the model’s performance in time series anomaly detection. Experiments on multivariate datasets, such as MSL, SMAP, and SMD, produced mixed results when absolute positional encoding (APE) or learnable PE was applied. Notably, the vanilla Transformer encoder achieved the highest F1 scores without any form of PE, as shown in Table 6. This indicates that preserving the temporal context in its original form may be more effective for reconstruction-based anomaly detection tasks.

Reconstruction tasks often rely on maintaining the integrity of the temporal context. Adding positional information can cause the model to differentiate features unnecessarily, potentially leading to overfitting on anomalous data. For univariate datasets, such as ECG, Gait, and RESP (see Table 7), the exclusion of PE also resulted in higher affiliation F1 scores. This aligns with the hypothesis that PE may introduce additional complexity, which is counterproductive for models focused on reconstructing temporal patterns.

Overall, these findings underscore that positional encoding is not essential for the vanilla Transformer encoder in this task. Instead, direct modeling of temporal dependencies can yield better results.

#### 4.5.3. Preprocessing Time Series Data

Preprocessing strategies played a crucial role in determining the model’s performance. The experiments examined various preprocessing combinations of normalization at both the dataset and sub-dataset levels, including min-max normalization and z-score normalization. The results in Table 8 and Table 9 provide the following key insights: For all the multivariate datasets, the highest F1 scores are achieved without using any normalization methods at the sub-dataset-level and dataset-level normalizations. For the univariate datasets, different normalization methods at sub-dataset-level and dataset-level normalizations produced the best affiliation F1 scores.

Choosing the preprocessing techniques like normalization that align with the specific characteristics of a dataset is crucial for preserving the integrity of its temporal structure. Proper preprocessing strategies for multivariate and univariate datasets enhance the model’s ability to distinguish normal patterns from anomalies, thereby improving reconstruction accuracy. These findings advocate for a tailored approach to preprocessing in time series anomaly detection, emphasizing its significant impact on model performance.

### 4.6. Hyperparameter Sensitivity

**Figure 2:**
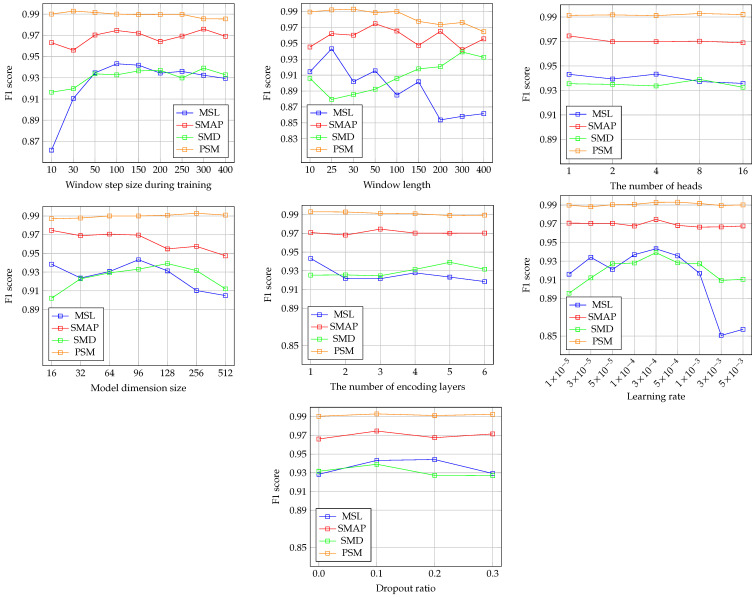
Hyperparameter sensitivity analysis results of seven key hyperparameters for our proposed framework on four multivariate datasets. The hyperparameters are training window step size, window length, the number of attention heads, model dimension size, the number of encoding blocks, learning rate, and dropout.

We performed a hyperparameter sensitivity analysis for the key hyperparameters of our proposed framework. Figure 2 presents the results of this analysis.

For the training window step size, the results indicate that the framework demonstrates robust performance within a specific range. However, performance declines significantly as the step size decreases, especially in the MSL and SMD datasets. This drop may result from variations in the time series segments generated according to the training window step size.

Regarding the window length, the framework exhibits high sensitivity to this hyperparameter. For the MSL and SMAP datasets, performance tends to decrease as the window length increases. Conversely, in the SMD dataset, shorter window lengths lead to poorer performance. Additionally, in the SMAP dataset, performance declines when the window length deviates from 50, either shorter or longer. Since window length directly affects the generation of training and testing time series segments, it is a critical hyperparameter. Given that the optimal window length varies across datasets, selecting an appropriate value is crucial for achieving the best results.

For the number of heads in the Transformer-based encoder, this hyperparameter appears to have minimal impact on model performance. While employing multiple attention heads allows the model to capture diverse dependencies, the results suggest that increasing the number of heads does not enhance performance. This indicates that effective anomaly detection can be achieved with a smaller number of dependencies.

The model dimension size shows varying effects depending on the dataset. For MSL and SMAP, performance decreases as the model dimension size increases. In contrast, for SMD, performance deteriorates when the model dimension size is either smaller or larger than 128. In PSM, performance remains unchanged across all model dimension sizes. These results highlight the need to carefully tune the model dimension size for optimal performance.

The impact of the number of encoder blocks varies depending on the dataset. For SMAP and PSM, performance remains almost unchanged regardless of the number of encoder blocks. In contrast, SMD and PSM exhibit greater performance fluctuations, indicating that this parameter can affect model performance in specific datasets.

The learning rate results show trends similar to the number of encoder blocks. In SMAP and PSM, performance is largely unaffected by changes in the learning rate. However, in SMD and PSM, significant variations in performance are observed, indicating that the learning rate is also an important hyperparameter.

Lastly, the dropout ratio results suggest that the framework achieves robust performance across different dropout ratios. Even in MSL, where the largest performance variation is noted, the changes are relatively minor.

## 5. Discussion

This work highlights the potential of a vanilla Transformer encoder with carefully selected design choices for unsupervised time series anomaly detection. Despite its simplicity, the proposed framework delivers performance that is competitive with, and in some cases exceeds, state-of-the-art models across various benchmark datasets. Notably, our framework achieved overall performance comparable to the SOTA models, AnomalyLLM [35], which relies on abnormal data injection [50,53,54]—a technique that synthesizes abnormal data from normal data during training. In contrast, our framework uses only normal data for training. These findings underscore the viability of a vanilla Transformer encoder-based model as an effective model for unsupervised time series anomaly detection.

In Transformer-based unsupervised time series anomaly detection, existing research has focused on enhancing architectural aspects of vanilla Transformer encoder while simultaneously incorporating various design choices. Anomaly Transformer [33] introduced a prior-association branch based on an adjacent-concentration inductive bias to enhance the distinction between normal and anomalous data. This additional branch enables the Transformer encoder to learn temporal dependencies that are easier to capture in normal data but more challenging for anomalous data, thereby improving overall model performance. Beyond architectural improvements, Anomaly Transformer [33] also explored design choices, such as time series segmentation with adjusted window length and segment embedding method along with positional embedding method.

DCdetector [39] employed a two-tower architecture that leverages multi-head attention in Transformer encoders to extract both patch-wise and in-patch association representations. This architecture differentiates normal and anomalous data by ensuring consistency between association representations derived from two distinct views of normal data. In addition to architectural enhancements, DCdetector [39] adopted several design choices, including time series segmentation, segment-level normalization to address non-stationarity, and segment embedding with positional encoding.

MEMTO [34] incorporated a gated memory module to store and leverage prototypical normal features – representative representations extracted from normal data – to facilitate the reconstruction of normal data while making the reconstruction of anomalous data more challenging. This module was integrated into a Transformer encoder as an architectural improvement. Furthermore, MEMTO [34] explored several design choices, such as time series segment generation with adjusted window length, time series segment embedding along with positional encoding, and the use of an asymmetric autoencoder to enhance feature learning.

AnomalyLLM [35] employed a teacher-student framework that leverages a pretrained large language model (LLM) with strong representational capabilities to perform representation learning, effectively distinguishing normal and abnormal data representations. It applies synthetic distortions to normal data to generate pseudo-anomalous data while utilizing a prototypical Transformer encoder to remove irrelevant information and extract informative features. Also, AnomlayLLM [35] incorporated several design choices, such as segment-level normalization to mitigate the non-stationarity, time series segment embedding along with position encoding, and an asymmetric encoder-decoder structure.

While prior studies have explored architectural enhancements of Transformer encoders alongside various design choices, their primary contributions emphasize architectural modifications rather than the design choices themselves. In contrast, our study demonstrates that effective anomaly detection can be achieved solely through carefully structured design choices, without modifying the Transformer encoder’s architecture when integrated into an asymmetric encoder-decoder framework utilizing a linear decoder.

Additionally, the prior studies have not explicitly stated certain design choices, such as data preprocessing techniques that significantly impact model performance. In contrast, our study clearly specifies all considered design choices and has conducted an ablation study to evaluate their individual impact on anomaly detection performance. We believe this work offers valuable insights for the development of time series anomaly detection models.

While our proposed framework demonstrated promising results, some limitations remain. First, unsupervised time series anomaly detection is inherently challenging due to the absence of abnormal data in a training dataset [17,33,34,35,39,49,50]. With only normal data available for training, it becomes difficult to optimize hyperparameters, like the threshold θ in Equation (11), that are sensitive to abnormal data.

Second, our evaluation of multivariate datasets in the experiments was conducted using the point adjustment (PA) method [42], which is known to potentially overestimate model performance [43,44]. To ensure fair comparisons with other models, we applied the same PA method as used in prior studies. However, for univariate datasets, we employed affiliation metrics [45], which are independent of the PA method, to provide a more reliable assessment of our framework’s performance in comparison to other models.

Third, we evaluated our proposed framework using a non-overlapping window evaluation protocol, where the window step size matches the window length. All the compared methods followed the same protocol for performance evaluation. In addition to the non-overlapping window protocol [17,33,34,35,39], some studies use overlapping window evaluation protocols. For example, a step size of 1 is employed in certain methods [23,24,30,55,56,57]. These differences in window step sizes during evaluation make direct comparisons across studies more challenging.

Lastly, we found that adjusting the window step size during training proved effective. However, keeping the step size fixed during the generation of time series segments restricts the model’s ability to capture the diverse temporal features present in time series data. This limitation may have adversely contributed to the significant performance gap observed compared to AnomalyLLM [35] on certain univariate datasets.

Future research should address the limitations identified in this study. First, the reliance on training datasets containing only normal data restricts the model’s ability to generalize to diverse anomaly patterns. Incorporating semi-supervised or self-supervised learning techniques could improve the model’s capacity to handle unseen anomalies. Additionally, the evaluation methodology, which relies on the point adjustment method and non-overlapping window protocols, could be enhanced by exploring alternative metrics and approaches to ensure more comprehensive and equitable model comparisons. Investigating dynamic or adaptive windowing strategies may further enhance the detection of temporal patterns. Lastly, extending the framework to accommodate more diverse datasets and application scenarios, including resource-constrained environments, could improve its practicality and scalability.

## 6. Conclusions

This study revisited the potential of a vanilla Transformer encoder for unsupervised time series anomaly detection. We demonstrated that when paired with a linear layer decoder and thoughtfully selected design choices, a vanilla Transformer encoder can achieve performance comparable to or even surpass state-of-the-art models across various univariate and multivariate benchmark datasets.

Our findings underscore the pivotal role of key design choices, such as segment-level normalization and denormalization, the omission of positional encoding, and precise hyperparameter tuning, in the model’s success. Despite its simplicity, the proposed asymmetric autoencoder framework, built on a vanilla Transformer encoder, effectively captures temporal dependencies for time series anomaly detection, emphasizing the often-underestimated potential of vanilla Transformer encoders.

## Figures and Tables

**Table 3:** F1 score results on the six multivariate datasets. **Bold values** indicate the best performance, and underlined values denote the second-best.

	MSL	SMAP	SMD	PSM	GECCO	SWAN-SF
Anomaly Transformer [33]	0.936	0.967	0.923	0.979	-	-
DCdetector [39]	**0.966**	0.970	0.872	0.979	0.466	0.734
MEMTO [34]	0.944	0.966	0.935	0.983	-	-
AnomalyLLM [35]	0.956	0.965	**0.958**	**0.997**	0.620	**0.804**
Ours	0.943	**0.975**	0.939	0.993	**0.920**	0.801

**Table 4:** Affiliation F1 score results on nine univariate datasets. **Bold values** indicate the best performance, and underlined values denote the second-best.

	ABP	Acceleration	Air Temperature	ECG	EPG
TS-TCC [49]	0.754	0.549	0.969	0.784	0.931
THOC [17]	0.815	0.776	0.971	0.760	0.908
NCAD [50]	0.794	0.849	0.758	0.735	0.789
AnomalyLLM [35]	**0.920**	0.956	**0.974**	0.787	**0.933**
Ours	0.838	**0.966**	0.972	**0.802**	0.840
	**Gait**	**NASA**	**Power Demand**	**RESP**	**Average**
TS-TCC [49]	0.794	0.511	0.763	0.560	0.735
THOC [17]	0.784	0.896	0.775	0.389	0.786
NCAD [50]	0.858	0.861	0.723	0.613	0.776
AnomalyLLM [35]	**0.871**	**0.961**	**0.886**	0.736	**0.892**
Ours	0.867	0.926	0.865	**0.763**	0.871

**Table 5:** Performance results of anomaly detection models across eight evaluation metrics. Aff. Precision and Aff. Recall refer to affiliation Precision and affiliation Recall, respectively, while R-AUC-ROC and R-AUC-PR denote Range-AUC-ROC and Range-AUC-PR. **Bold values** indicate the best performance for each evaluation metric within each benchmark.

		Accuracy	F1 Score	Aff. Precision	Aff. Recall	R-AUC-ROC	R-AUC-PR	VUS-ROC	VUS-PR
MSL	Anomaly Transformer [33]	0.987	0.939	0.518	0.960	0.900	0.879	0.882	0.863
	DCdetector [39]	**0.991**	**0.966**	0.518	**0.974**	0.932	**0.916**	0.932	**0.917**
	Ours	0.988	0.943	**0.617**	0.955	**0.957**	0.880	**0.957**	0.880
SMAP	Anomaly Transformer [33]	0.991	0.964	0.514	**0.987**	**0.963**	0.941	0.955	0.934
	DCdetector [39]	0.992	0.970	0.515	0.986	0.960	**0.942**	0.952	**0.935**
	Ours	**0.993**	**0.945**	**0.591**	0.885	0.956	0.897	**0.956**	0.895
PSM	Anomaly Transformer [33]	0.987	0.974	0.554	0.803	0.918	0.930	0.887	0.907
	DCdetector [39]	0.990	0.979	0.547	0.829	0.916	0.929	0.884	0.906
	Ours	**0.996**	**0.993**	**0.774**	**0.927**	**0.972**	**0.958**	**0.959**	**0.950**

**Table 6:** Ablation analysis results of segment-level normalization-denormalization and positional encoding on three multivariate datasets. SegND represents segment-level normalization-denormalization, APE represents absolute positional encoding, and numbers in the cells represent F1 scores. **✗** signifies that the corresponding one is not used, while **✓** indicates that the corresponding one is used. **Bold values** indicate the best performance.

SegND	Positional Encoding	MSL	SMAP	SMD
**✗**	**✗**	0.909	0.740	0.830
**✗**	APE (sinusoid)	0.846	0.734	0.794
**✗**	APE (learnable)	0.902	0.710	0.845
**✓**	**✗**	**0.943**	**0.975**	**0.939**
**✓**	APE (sinusoid)	0.914	0.966	0.904
**✓**	APE (learnable)	0.924	0.957	0.923

**Table 7:** Ablation analysis results of segment-level normalization-denormalization and positional encoding on three univariate datasets. SegND, APE, **✗** and **✓** are described in Table 6. The numbers in the cells represent affiliation F1 scores. **Bold values** indicate the best performance.

SegND	Positional Encoding	ECG	Gait	RESP
**✗**	**✗**	0.739	0.783	0.693
**✗**	APE (sinusoid)	0.772	0.768	0.702
**✗**	APE (learnable)	0.746	0.819	0.703
**✓**	**✗**	**0.802**	**0.867**	**0.763**
**✓**	APE (sinusoid)	0.752	0.820	0.729
**✓**	APE (learnable)	0.759	0.850	0.721

**Table 8:** Ablation analysis results of preprocessing time series data on three multivariate datasets, each comprising multiple sub-datasets (see Table 1). Sub-Dataset-Level refers to a case where a normalization method is applied to each sub-dataset within a dataset, while Dataset-Level refers to a case where a normalization method is applied to a single dataset that concatenates all its sub-datasets. The numbers in the cells represent F1 score results. **✗** signifies that the corresponding one is not used, while **✓** indicates that the corresponding one is used. **Bold values** indicate the best performance.

Sub-Dataset-Level	Dataset-Level	MSL	SMAP	SMD
**✗**	**✗**	**0.943**	**0.975**	**0.939**
**✗**	min-max	0.919	0.925	0.931
**✗**	z-score	0.887	0.707	0.877
min-max	**✗**	0.611	0.842	0.812
min-max	min-max	0.605	0.830	0.807
min-max	z-score	0.750	0.640	0.740
z-score	**✗**	0.923	0.692	0.811
z-score	min-max	0.922	0.823	0.808
z-score	z-score	0.887	0.655	0.799

**Table 9:** Ablation analysis results of preprocessing time series data on four univariate datasets, each comprising multiple sub-datasets (see Table 2). Sub-Dataset-Level and Dataset-Level are described in Table 8. The numbers in the cells represent affiliation F1 scores. **✗** signifies that the corresponding one is not used, while **✓** indicates that the corresponding one is used. **Bold values** indicate the best performance.

Sub-Dataset-Level	Dataset-Level	ECG	EPG	Gait	NASA
**✗**	**✗**	0.672	**0.840**	0.725	**0.926**
**✗**	min-max	0.657	0.829	0.709	0.872
**✗**	z-score	0.670	0.812	0.712	0.892
min-max	**✗**	0.792	0.723	**0.867**	0.846
min-max	min-max	0.779	0.726	0.850	0.862
min-max	z-score	**0.802**	0.707	0.857	0.850
z-score	**✗**	0.735	0.667	0.825	0.914
z-score	min-max	0.756	0.677	0.815	0.883
z-score	z-score	0.764	0.711	0.831	0.870

## Data Availability

We have used the benchmarks which are publicly available.

## Abbreviations

The following abbreviations are used in this manuscript:

1D	one-dimensional
ABP	Arterial blood pressure benchmark
APE	Absolute Positional Encoding
CNN	Convolutional Neural Network
ECG	Electrocardiogram benchmark
EPG	Electrical Penetration Graph benchmark
GECCO	Genetic and Evolutionary Computation Conference benchmark
GRU	Gated Recurrent Unit
LSTM	Long Short-Term Memory
MSL	Mars Science Laboratory benchmark
PA	Point Adjustment
PE	Positional Encoding
PSM	Pooled Server Metrics benchmark
RESP	Respiration benchmark
RevIN	Reversible Instance Normalization
RNN	Recurrent Neural Network
SegND	Segment-level Normalization-Denormalization
SMAP	Soil Moisture Active Passive satellite benchmark
SMD	Server Machine Dataset benchmark
SWAN-SF	Space Weather Analytics for Solar Flares benchmark
TCN	Temporal Convolutional Network

## Appendix A

**Table A1:** Hyperparameter configurations on multivariate datasets.

	MSL	SMAP	SMD	PSM	GECCO	SWAN-SF
# epochs	1	1	6	4	4	5
Batch size	32	32	32	32	32	32
# layers	1	3	5	2	2	2
# heads	1	1	8	8	8	8
Model dimension size	96	16	128	256	128	512
Feed-forward dimension size	96	32	256	512	256	1024
Window length	25	50	300	30	10	400
Training window stride	100	100	300	30	10	200
Test window stride	25	50	300	30	10	400
Dropout ratio	0.1	0.1	0.1	0.1	0.1	0.0
Learning rate	3 × $10^{-4}$	3 × $10^{-4}$	3 × $10^{-4}$	3 × $10^{-4}$	3 × $10^{-4}$	3 × $10^{-4}$
Sub-dataset-level norm	-	-	-	-	-	-
Dataset-level norm	-	-	-	-	z-score	-

**Table A2:** Hyperparameter configurations of univariate datasets.

	ABP	Acceleration	Air Temperature	ECG	EPG	Gait	NASA	Power Demand	RESP
# epochs	3	5	10	1	3	2	5	2	1
Batch size	32	32	32	32	32	32	8	32	32
# layers	2	2	1	2	2	2	2	3	1
# heads	8	8	8	8	8	8	1	8	8
Model dimension size	512	256	256	256	256	256	32	128	256
Feed-forward dimension size	1024	512	512	512	512	512	32	256	512
Window length	100	200	50	100	30	350	100	100	100
Training window stride	100	400	50	100	30	350	100	100	100
Test window stride	100	200	50	100	30	350	100	100	100
Dropout ratio	0.0	0.1	0.1	0.1	0.1	0.1	0.1	0.0	0.1
Learning rate	5 × $10^{-5}$	3 × $10^{-5}$	3 × $10^{-3}$	3 × $10^{-4}$	5 × $10^{-5}$	5 × $10^{-5}$	5 × $10^{-5}$	3 × $10^{-3}$	3 × $10^{-3}$
Sub-dataset-level norm	minmax	minmax	-	minmax	minmax	minmax	-	minmax	minmax
Dataset-level norm	z-score	z-score	-	z-score	-	-	-	z-score	z-score

**Table A3:** Detailed performance evaluation results on six multivariate time series datasets. **Bold values** indicate the best performance, and underlined values denote the second-best.

	MSL			SMAP			SMD			PSM			GECCO			SWAN-SF		
	**P**	**R**	**F1**	**P**	**R**	**F1**	**P**	**R**	**F1**	**P**	**R**	**F1**	**P**	**R**	**F1**	**P**	**R**	**F1**
Anomaly Transformer	0.921	0.952	0.936	0.942	0.994	0.967	0.894	0.955	0.923	0.969	0.989	0.979	-	-	-	-	-	-
DCdetector	0.937	0.997	**0.966**	0.956	0.989	0.970	0.836	0.911	0.872	0.971	0.987	0.979	0.383	0.597	0.466	0.955	0.596	0.734
MEMTO	0.921	0.968	0.944	0.938	0.996	0.966	0.891	0.984	0.935	0.975	0.992	0.983	-	-	-	-	-	-
AnomalyLLM	0.937	0.979	0.958	0.944	0.969	0.956	0.934	0.998	**0.965**	0.996	0.998	**0.997**	0.511	0.793	0.620	0.873	0.745	**0.804**
Ours	0.917	0.971	0.943	0.964	0.986	**0.975**	0.894	0.989	0.939	0.992	0.994	0.993	0.919	0.921	**0.920**	0.861	0.749	0.801

**Table A4:** Affiliation precision, affiliation recall, and affiliation F1 score results for our model and other comparisons on nine univariate time series anomaly detection datasets. **Bold values** represent a top result and underline values denote a second result on a metric.

	ABP			Acceleration			Air Temperature			ECG			EPG		
	**AP**	**AR**	**AF1**	**AP**	**AR**	**AF1**	**AP**	**AR**	**AF1**	**AP**	**AR**	**AF1**	**AP**	**AR**	**AF1**
TS-TCC	0.763	0.745	0.754	0.555	0.543	0.549	0.980	0.957	0.969	0.758	0.782	0.784	0.928	0.935	0.931
THOC	0.822	0.808	0.815	0.782	0.770	0.776	0.984	0.958	0.971	0.762	0.758	0.760	0.911	0.905	0.908
NCAD	0.802	0.786	0.794	0.855	0.842	0.849	0.762	0.747	0.758	0.737	0.732	0.735	0.795	0.783	0.789
AnomalyLLM	0.931	0.910	**0.920**	0.965	0.948	0.956	0.989	0.959	**0.974**	0.768	0.808	0.787	0.935	0.932	**0.933**
Ours	0.725	0.991	0.838	0.934	1.000	**0.966**	0.951	0.993	0.972	0.698	0.942	**0.802**	0.743	0.966	0.840
	**Gait**			**NASA**			**Power Demand**			**RESP**			**Average**		
	**AP**	**AR**	**AF1**	**AP**	**AR**	**AF1**	**AP**	**AR**	**AF1**	**AP**	**AR**	**AF1**	**AP**	**AR**	**AF1**
TS-TCC	0.798	0.790	0.794	0.512	0.508	0.511	0.767	0.759	0.763	0.561	0.560	0.560	0.736	0.731	0.735
THOC	0.788	0.780	0.784	0.902	0.891	0.896	0.777	0.772	0.775	0.382	0.395	0.389	0.790	0.782	0.786
NCAD	0.864	0.852	0.858	0.869	0.853	0.861	0.724	0.723	0.723	0.613	0.612	0.613	0.780	0.770	0.776
AnomalyLLM	0.891	0.852	**0.871**	0.969	0.953	**0.961**	0.888	0.884	**0.886**	0.736	0.736	0.736	0.897	0.887	**0.892**
Ours	0.767	0.998	0.867	0.892	0.962	0.926	0.766	0.994	0.865	0.629	0.969	**0.763**	0.789	0.979	0.871
